# Macular Buckling Surgery for Retinal Detachment Associated with Macular Hole in High Myopia Eye

**DOI:** 10.4274/tjo.55453

**Published:** 2017-12-25

**Authors:** Kenan Sönmez, Ali Keleş

**Affiliations:** 1 University of Health Sciences, Ulucanlar Eye Training and Research Hospital, Ophthalmology Clinic, Ankara, Turkey

**Keywords:** High Myopia, retinal detachment, macular hole, macular buckle

## Abstract

A 68-year-old woman presented to our clinic with a 1-month history of central scotoma and visual loss in her right eye. The best corrected visual acuity (BCVA) was hand motion in her right eye. Fundus examination showed myopic chorioretinal degeneration in association with posterior staphyloma and the retina was slightly elevated throughout the macula. Optical coherence tomography (OCT) revealed retinal detachment involving the posterior pole with a macular hole and staphyloma. The patient underwent pars plana vitrectomy, internal limiting membrane peeling, macular buckling, and perfluoropropane gas tamponade. At 3-month follow-up, her BCVA was improved to counting fingers at 1 meter and flattened retina with closed macular hole was observed by OCT. Myopic macular hole with retinal detachment associated with posterior staphyloma represent a challenge regarding their management and several surgical techniques have been described. Although satisfactory anatomical improvement is achieved in these eyes after surgery, the visual acuity outcomes may be poorer than expected due to the chorioretinal atrophy at the posterior pole.

## INTRODUCTION

Although macular hole is reported to be a rare cause of retinal detachment (RD), accounting for approximately 0.5% of all detachment cases, this figure has been reported as 9% and over in some races.^1,2^ One of the most common causes of macular holes leading to RD is high myopia.^1^ Although the pathogenesis is not fully understood, various mechanisms have been suggested to play a role in the development of RD associated with macular hole (MHRD) in high myopic patients. These include increased vitreous traction due to posterior staphyloma, reduced chorioretinal adhesion due to posterior chorioretinal atrophy, stiffening of the internal limiting membrane (ILM), increased tension in retinal vessels, and tangential forces created by increased cortical vitreous contractions.^3,4^

The treatment of MHRD in high myopia is difficult. Several surgical approaches have been recommended, such as pneumoretinopexy, pars plana vitrectomy (PPV) with ILM peeling or macular buckling (MB). In this study, we present the outcomes of PPV, ILM peeling, MB, and perfluoropropane (C_3_F_8_) gas tamponade performed to treat MHRD in a patient with high myopia and posterior staphyloma.

## CASE REPORT

**Figure 1:**
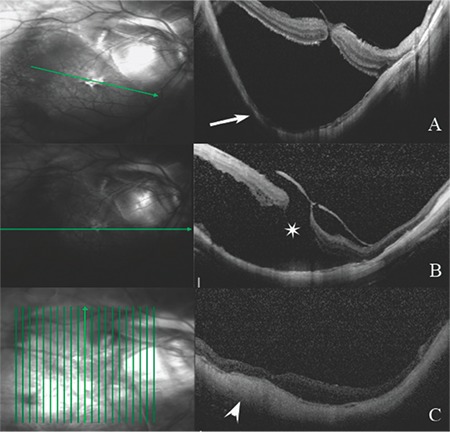
(A) Preoperative optical coherence tomography shows posterior staphyloma in the right eye (arrow) and (B) retinal detachment associated with a full-thickness macular hole (star). (C) Postoperative optical coherence tomography shows closure of the macular hole, retinal attachment, and reduced posterior staphyloma (arrowhead)

**Figure 2:**
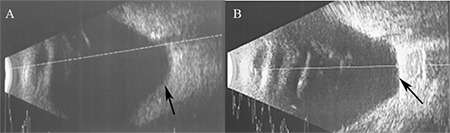
On B-mode ultrasonography, (A) a bulge is observed in the posterior staphyloma area preoperatively (arrow); (B) postoperatively, the posterior staphyloma is flattened due to pressure exerted by the explant (arrow)

A 68-year-old female patient presented with complaints of low vision and central vision loss in her right eye for the past month. Her best corrected visual acuity was hand motion in both eyes. Intraocular pressure was 19 mmHg in the right eye and 17 mmHg in the left eye. Slit-lamp examination revealed bilateral nuclear sclerosis. On fundus examination, bilateral posterior staphyloma with myopic degenerative changes were observed, as well as a shallow RD associated with the posterior staphyloma in the right eye. Examination by optical coherence tomography (OCT) showed RD associated with the full-thickness macular hole in the center of the posterior staphyloma of the right eye (Figure 1A and Figure 1B). Anterior-posterior axis length was 33.65 mm. B-mode ultrasonography showed significant posterior bulging of the sclera (Figure 2A).

**Figure 3:**
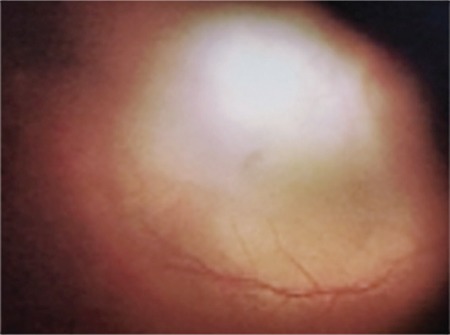
Intraoperative color fundus photograph shows how the transillumination method was used to determine where the explant presses on the posterior pole

Surgical repair was done by dissecting the conjunctiva and Tenon’s capsule in an approximately 150-160 degree area of the superotemporal region of the right eye, and bridle sutures were passed through the superior and lateral rectus. In the superotemporal region, 5/0 nylon sutures were placed in the sclera approximately 20 mm from the limbal zone where the implant would be fixed between the insertion points of the superior and inferior oblique muscles. Following phacoemulsification and intraocular lens implantation in the posterior chamber, triamcinolone acetonide (TA)-assisted PPV and ILM peeling were performed. Before securing the explant (AJL Ophthalmic) to the superotemporal region, a fiber-optic light attached to the explant was used to check where the explant contacted the posterior pole by transillumination (Figure 3). Laser photocoagulation was applied to the hole and degenerative areas in the peripheral retina, followed by fluid-gas exchange using C_3_F_8_.

The patient was recommended to lie in prone position for 3 days postoperatively. Fundus examination and B-mode ultrasonography performed at postoperative 2 months revealed a bulge in the macular area associated with the local explant (Figure 2B). At postoperative 3 months, the patient’s visual acuity was counting fingers from 1 meter. Fundus examination showed that the macular hole had closed and the retina was attached. These findings were confirmed with OCT (Figure 1C).

## DISCUSSION

The treatment of MHRD in patients with high myopia presents a considerable challenge. Several surgical approaches have been suggested for such cases. Since 1982, PPV has generally been accepted as the preferred surgical approach for the treatment of MHRD high-myopic eyes.^5^ Using TA during PPV facilitates the detection of vitreous cortex remnants and the differentiation and visualization of the epiretinal membrane. Compared to TA-assisted procedures, patients undergoing PPV without TA show a higher rate of repeat surgery due to postoperative development of preretinal fibrosis.^6^ ILM peeling eliminates the risk of prefoveal vitreous cortex remnants following PPV. Moreover, ILM peeling with PPV improves the chances of surgical success by reducing the amount of tangential traction at the macular hole.^7^ In light of these data, we also applied TA-assisted PPV with ILM peeling in our case. Previous studies have reported anatomical success rates of 70-92% with PPV, ILM peeling, and gas tamponade in the treatment of MHRD in high myopic eyes.^8,9,10^ However, although PPV with ILM peeling and gas tamponade is the primary surgical approach for such cases, it may not be adequate to address certain pathophysiological factors such as the tension created by the posterior staphyloma. The presence of posterior staphyloma in these patients may lead to complications such as foveoschisis, foveal detachment, and MHRD. Morita et al.^3^ showed that the incidence of RD in eyes with macular holes was associated with degree of myopia, chorioretinal changes, and the presence of posterior staphyloma. Wei et al.^11^ reported that greater axial length, severe chorioretinal atrophy, and posterior staphyloma negatively affected postoperative anatomic success in high myopia patients with MHRD. Therefore, MB methods have been proposed to prevent increased tension due to posterior staphyloma.

MB is an old surgical technique used to counteract the pulling effect of the staphyloma.^12^ However, it is difficult to accurately position the material during the procedure so that it will have the desired effect on the macula. The second difficulty we have with this procedure is the availability of explants. Various materials such as silicone sponge, silicon-coated polymethylmethacrylate, silicon plate containing metal wire (Ando), and polytetrafluoroethylene are used as explants in MB. Theodossiadis and Theodossiadis^13^ reported achieving anatomic success in 88% of patients with high myopia and MHRD using MB with silicone sponges. Numerous studies have reported anatomical success rates of 90% or higher after MB in cases with MHDR.^14,15^ These high rates of reported anatomical success in high-myopic MHRD patients have led to MB gaining prominence, especially when treating patients with posterior staphyloma.

By flattening the excessive concavity in the posterior pole caused by the posterior staphyloma, MB reduces the anterior-posterior traction caused by both the posterior staphyloma itself and the tension in the retinal arteries. However, PPV and ILM peeling applied in addition to MB may be effective in preventing the recurrence that is sometimes seen in these cases. PPV and ILM peeling eliminates tangential and centripetal traction which can result from ILM and epiretinal membrane. Therefore, combined surgical approaches have been proposed to increase both anatomic success and the likelihood of macular hole closure. Alkabes et al.^16^ reported that a combination of PPV, ILM peeling, and MB resulted in macular hole closure in 81% and retinal reattachment in 95% of MHRD cases. In the same study,^16^ this combined procedure led to macular hole closure in 57% and retinal reattachment in 90.5% of patients who had not responded well to previous surgical approaches. Similarly, in a large prospective study by Ma et al.^17^ comparing the outcomes of PPV with ILM peeling and combined PPV, ILM peeling, and MB in patients with MHRD, the combined procedure was associated with significantly higher rates of both macular hole closure and retinal reattachment. We also performed PPV, ILM peeling, MB, and gas tamponade procedures in our MHRD patient due to findings of increased axial length, posterior staphyloma, and chorioretinal atrophy. We observed both macular hole closure and retinal reattachment postoperatively. However, there was not as much functional improvement as we expected, and the increase in visual acuity was limited. Even if no intraoperative complications are noted, vascular structures or optic nerve damage may occur while the explant is placed. In addition, both the preexisting RD and the thin, delicate retinas in eyes with high myopia and MHRD can cause serious complications during ILM peeling, such as the formation of new holes in the retina. Nevertheless, we observed no intraoperative or postoperative complications related to ILM peeling in our case.

The combination of PPV, ILM peeling, MB, and gas tamponade may be effective in patients with high myopia and MHRD. However, although the anatomical success is high with this procedure, functional success may be limited due to chorioretinal atrophy resulting from high myopia. In our patient, limited functional improvement was achieved due to chorioretinal atrophy in the macular region. Therefore, the fact that the severity of chorioretinal atrophy in the posterior pole will limit functional success should be considered prior to surgical intervention in these patients.
